# High and low molecular weight hyaluronic acid-coated gold nanobipyramids for photothermal therapy

**DOI:** 10.1039/c7ra11667e

**Published:** 2018-02-28

**Authors:** Shuang Zhao, Ying Tian, Wenfei Liu, Yunyan Su, Yunlei Zhang, Zhaogang Teng, Ying Zhao, Shouju Wang, Guangming Lu, Zhenghong Yu

**Affiliations:** Department of Medical Oncology, Jinling Hospital, Medical School of Nanjing University Nanjing 210002 P. R. China m_fish@189.cn; Department of Medical Imaging, Jinling Hospital, Medical School of Nanjing University Nanjing 210002 P. R. China cjr.luguangming@vip.163.com shouju.wang@zoho.com; Key Laboratory for Organic Electronics and Information Displays, Institute of Advanced Materials (IAM), Jiangsu National Synergetic Innovation Centre for Advanced Materials (SICAM), Nanjing University of Posts & Telecommunications 9 Wenyuan Road Nanjing 210023 P. R. China

## Abstract

Triple-negative breast cancer (TNBC) is an aggressive subtype of breast cancer. It is known that hyaluronic acid (HA) binds CD44 receptors, which are overexpressed on the surface of TNBC cells. To optimize the targeting ability of HA, in this study we coated gold nanobipyramids (GBPs) with high and low molecular weight HA (380 kDa and 102 kDa), named GBPs@h-HA and GBPs@l-HA, respectively. GBPs@l-HA and GBPs@h-HA had excellent stability when dispersed in water and PBS (pH 7.4) for seven days. The HA density was calculated by the ratio of HA to GBPs@l-HA and GBPs@h-HA, which was 13.22 and 4.77, respectively. The two nanoparticles displayed good photostability, which was evaluated by their photothermal performance and similar biocompatibility. Inductively coupled plasma atomic emission spectrometry (ICP-AES) revealed superior cellular uptake of GBPs@h-HA over GBPs@l-HA. Upon 808 nm laser irradiation, the GBPs@h-HA also showed higher therapeutic efficacy than GBPs@l-HA both *in vitro* and *in vivo*. Overall, our study demonstrates that the molecular weight of HA plays an important role in the targeting ability and thus photothermal therapeutic efficacy of HA-coated gold nanobipyramids.

## Abstract 2

Triple-negative breast cancer (TNBC) is an aggressive subtype of breast cancer. Hyaluronic acid (HA) could bind CD44 receptors, which are overexpressed on the surface of TNBC cells. Upon 808 nm laser irradiation, the GBPs@HA showed high therapeutic efficacy *in vivo*.

## Graphical abstract

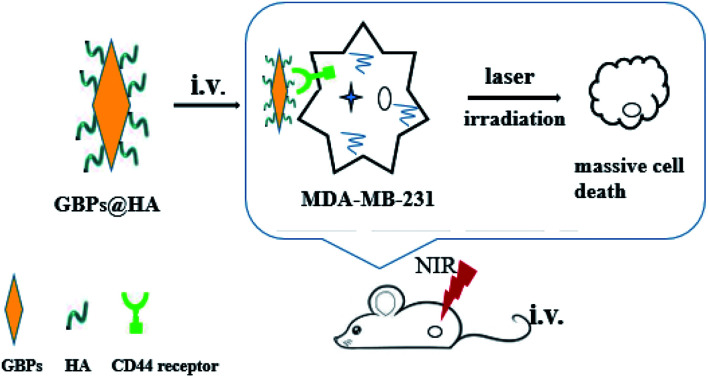

## 1 Introduction

Breast cancer is a fatal cancer disease in women and caused 40 000 deaths in the USA in 2015.^1–3^ Triple-negative breast cancer (TNBC) is characterized by a lack of the oestrogen receptor (ER), progesterone receptor (PR), or human epidermal growth factor receptor 2 (HER-2) and accounts for 15% of breast cancer diagnoses.^4^ TNBC is typically heterogeneous, aggressive and associated with a poor prognosis, which means it is less responsive to conventional therapeutic methods and lacks the well-defined surface markers necessary for effective targeted therapy.^4–6^ Currently, as the bottleneck of targeted therapy is the lack of TNBC-specific entities that can recognize TNBC cells from non-neoplastic cells, the mainstay of therapy for TNBC remains chemotherapy, to which most patients are highly resistant.^7,8^ Therefore, the identification of a feasible cancer-selective method, especially one that is directed towards targeted therapy for TNBC, is urgent.

CD44 receptors are mainly overexpressed in TNBC cells; 99.2% of MDA-MB-231 cells have CD44 receptors, and almost no HEK 293T cells have CD44 receptors.^9–12^ In recent years, CD44 and its variants have gained much attention for their utility as potential therapeutic targets and prognostic markers for cancer therapy.^13^ Xu *et al.* showed that higher expression levels of CD44 at the mRNA and protein levels are one of the diagnosing biomarkers of TNBC.^14^ As a major receptor of HA, CD44 has a high affinity with HA binding, which can drive numerous tumour-promoting signalling pathways and transporter activities *via* the HA–CD44 endocytosis pathway.^15–17^ As a potential targeting moiety, the molecular weight (MW) of HA, typically ranging from 50 kDa to 20 000 kDa, varies with its chain length.^18,19^ Wolny *et al.* measured the binding ability of HA with different molecular weights to CD44 and found that the binding between HA with a MW higher than 262 kDa and CD44 is strong and irreversible, while the binding between HA with a MW lower than 262 kDa is much weaker and reversible.^20^ It is of interest to note that only HA with a molecular weight lower than 6 kDa would enhance tumour angiogenesis. HA with a molecular weight higher than 6 kDa would inhibit angiogenesis.^21–23^ In previous studies of HA-coated liposomes, H. S. S. Qhattal *et al.* provided evidence that HA-liposomes were taken up into cells *via* CD44 receptor-controlled endocytosis, and the cellular uptake was increased when compared with PEG-liposomes.^24^ However, HA-liposomes showed a different cellular targeting efficiency, which depended strongly upon the HA MW and grafting density, indicating its varied binding affinity to the CD44 receptor.^24–26^

In this study, we selected two MWs of HA (380 and 102 kDa), respectively named as h-HA and l-HA, to investigate the targeting ability of different MWs of HA-coated gold nanobipyramids (GBPs) for TNBC.^21^ GBPs have raised much attention because of their superior optical properties, larger extinction cross section, and more significant local electric-field enhancement than gold nanorods.^27–29^ Different from the rounded ends of gold nanorods, GBPs have two sharper apexes rising onto the cross point.^30^ Researchers demonstrated that GPBs have been used as one kind of photothermal conversion agent for cancer ablation, which is attributed to their high absorbance in the near-infrared (NIR) window and approved biosafety in the human body.^31–33^ However, to enhance the therapeutic efficacy of photothermal therapy (PTT) for TNBC, it is essential to deliver the photothermal conversion agents to tumour selectively.^34–38^

To optimize the targeting ability of HA-coated GBPs, the obtained HA-coated GBPs, respectively named GBPs@h-HA and GBPs@l-HA, were assessed in terms of their biocompatibility, cellular uptake, toxicity and photothermal therapeutic efficacy *in vitro* and *in vivo*. The results revealed that GBPs coated with high molecular weight HA (MW 380 kDa) have a higher degree of cellular uptake and show enhanced photothermal therapeutic efficacy *in vivo* upon 808 nm laser irradiation.

## 2 Experimental

### Materials

Sodium borohydride (NaBH_4_) and *N*-cetyltrimethylammonium bromide (CTAB) were purchased from Sinopharm Chemical Reagent Co., Ltd. (Shanghai, China). Hyaluronic acid (Mw 380 kDa (h-HA) and 102 kDa (l-HA)) was purchased from Freda (Jinan, China). Hydrogen tetrachloroaurate trihydrate (HAuCl_4_·3H_2_O), silver nitrate (AgNO_3_), l-ascorbic acid (l-AA), dimethyl sulphoxide (DMSO), heat-inactivated foetal bovine serum (FBS) and phosphate buffered saline (PBS) were purchased from Sigma-Aldrich (St. Louis, MO, USA). Hydrogen peroxide (H_2_O_2_), glacial acetic acid (CH_3_COOH), and ammonium hydroxide (NH_3_·H_2_O) were purchased from Aladdin Chemical. Hexadecyltrimethylammonium chloride (CTAC) was purchased from TCI (Shanghai, China). Acetic acid sodium salt trihydrate (CH_2_COONa·3H_2_O) was purchased from Nanjing Chemical Reagent Co., Ltd. (Nanjing, China).

3-(4,5-Dimethylthiazol-2-yl)-2,5-diphenyltetrazolium bromide (MTT) was bought from Keygen Biotech (Co. Ltd. China). Human embryonic kidney cell line (HEK 293T, a CD44 negative normal human embryonic kidney cell line) was obtained from ATCC (Manassas, VA). MDA-MB-231/Luc (breast cancer cell line with luciferase, a CD44 positive TNBC cell line)^10,39^ was purchased from Shanghai Bioray Biotechnology (Co. Ltd. China). Ultrapure water (resistivity 18.2 MΩ cm at 25 °C) was obtained from a Milli-Q system. All chemicals were of analytical grade and used as received without further treatment.

### The synthesis of GBPs@l-HA and GBPs@h-HA

#### Growth of bipyramids from the seeds

Gold nanobipyramids (GBPs) were prepared through seed-mediated growth.^40^ In brief, the gold seed solution was prepared by adding 0.125 mL of aqueous HAuCl_4_ (0.01 M) and 0.25 mL of the sodium citrate (0.01 M) into 9.625 mL of deionized water in sequence. Then, 0.15 mL of the cold freshly prepared NaBH_4_ (0.01 M) was rapidly added to the above solution while stirring at 14 000 rpm. The mixture was removed from the magnetic stirring apparatus as its colour turned from orange to light pink. After that, the gold seed was stewed for 2 h.

When 2 mL of aqueous HAuCl_4_ (0.01 M) was added to a solution of CTAB (40 mL, 0.1 M), the colour of the solution changed from neutral to ginger, and then AgNO_3_ (400 μL, 0.01 M) and hydrochloric acid (800 μL, 1 M) were added into the solution stepwise. The mixture solution turned from ginger to colourless with moderate shaking after 320 μL of freshly prepared l-AA (0.1 M) was added. Then, 200 μL of the prepared gold seed solution was added to the above solution under several vigorous up–down reversals. The prepared solution was stored in a water bath at 30 °C overnight.

#### Separation of the silver-growth

The purification of GBPs was completed through the following methods.^29^ Nanoparticles were collected by centrifugation at 10 000 × *g* for 25 min at room temperature. The residues were diluted in 20 mL of CTAC aqueous solution. Next, 600 μL of 0.1 M AgNO_3_ and 3 mL of 0.1 M freshly prepared l-AA were added in the abovementioned mixture solution. Finally, the mixture solution changed from purple to chocolate brown after being kept in a constant temperature oven for 4 h at 65 °C, and then the residues were collected by centrifugation at 10 500 × *g* for 25 min. Following these steps, 15 mL of deionized water and 10 mL of 0.5 M CTAC were added to dilute the residues, and the brown mixture was made as it proved overnight at 26–28 °C.

The supernatant and the precipitates apparently separated with each other after a night, and as a result, the excess supernatant was removed as much as possible. The precipitates were re-dispersed in approximately 20 mL of ultrapure water, and 400 μL of NH_3_·H_2_O and 180 μL of H_2_O_2_ was added.

#### Preparation of GBPs@l-HA and GBPs@h-HA

Twenty millilitres of the as-synthesized GBPs were collected by centrifugation and washed three times with fresh water to sufficiently remove the CTAB. The residue was dispersed in 10 mL of ultrapure water, and then the 10 mL of GBPs solution was added drop by drop in the prepared 20 mL of HA (l-HA or h-HA) (2 mg L^−1^) with stirring for 3 h at 800 rpm. Finally, hyaluronic acid-coated gold nanobipyramids were obtained after washing with ultrapure water three times at 6000 rpm for 20 min. In brief, the two end-products were named GBPs@h-HA and GBPs@l-HA.

### Characterization of GBPs, GBPs@l-HA, and GBPs@h-HA

The sizes and morphologies of the as-prepared GBPs, GBPs@l-HA, and GBPs@h-HA were recorded *via* a transmission electron microscope (TEM, JEM-200CX microscope, Japan). Ultraviolet-visible absorptions were recorded by a Lambda 35 UV-vis spectrophotometer (PerkinElmer Instruments, USA). The surface charges and the diameters of the samples in water were determined using a NanoBrook ZetaPlus Zeta Potential Analyzer (Brookhaven Instruments, USA). Nitrogen sorption isotherms were assessed by a Micromeritics ASAP 2020 V4.00 system (USA). The 808 nm diode-pumped laser source was obtained from Hi-Tech Optoelectronic Co., Ltd. (China). Infrared thermal images were recorded using an infrared camera (MAGNITY f15F1, Wuhan VST Light & Technology Co., Ltd., China). The amount of l-HA or h-HA conjugated to the GBPs was detected by CTAB precipitation assay.^25^ Briefly, 0.05 mL of 0.2 M sodium acetate buffer was added in the 96-well plate to incubate with 0.05 mL of HA standard solutions (0.125–2 mg mL^−1^) or diluted supernatants for 10 min at 37 °C. Then, 0.1 mL of 0.01 M CTAB solution was added, and the absorbance of the mixture was obtained at a wavelength of 570 nm using a Lambda 35 UV-vis spectrophotometer (PerkinElmer Instruments, USA) within 10 minutes. The amount of grafted HA was calculated by the initial amount (2 mg mL^−1^) of HA minus the amount in the supernatant fraction (calculated by the standard curve of l-HA or h-HA), and the mass ratio was calculated by the HA to GBPs@l-HA or GBPs@h-HA ratio.

#### Cell culture

The human breast cancer cell line MDA-MB-231/Luc was used *in vitro*, and the culture conditions were as follows: Minimum Essential Medium (MEM, GIBCO, USA), 10% Foetal Bovine Serum (FBS, GIBCO, USA), 1% MEM Non-Essential Amino Acids (MEM NEAA, GBICO, USA), 1% sodium pyruvate (GBICO, China), and 1% penicillin–streptomycin (GBICO, USA). HEK 293T cells were cultured in RPMI Medium 1640 (GBICO, USA) containing 10% Foetal Bovine Serum (FBS, GIBCO, USA). Both kinds of cells were cultured in a humidified incubator at 37 °C with 5% CO_2_. All nanoparticles were carefully washed with ultrapure water three times. All the results were repeated more than 3 times. The cell morphology was captured using an Olympus microscope (Olympus IX71, Tokyo, Japan).

### Cellular uptake evaluation by inductively coupled plasma (ICP) analysis

In the ICP analysis, we incubated 15 mg L^−1^ of GBPs@l-HA or GBPs@h-HA with MDA-MB-231/Luc cells in MEM. The cultured cells were washed with PBS twice and harvested with 0.05% trypsin–EDTA (GIBCO, USA) before being resuspended in 3 mL of PBS. A volume of approximately 400 μL of each of the mixtures was used for protein quantitative analysis using the Bradford assay (Bio-Rad, USA). The remaining amounts were centrifuged and digested with 2 mL of high nitric acid and 2 mL of fresh aqua regia in a stepwise manner to determine the amount of gold *via* an inductively coupled plasma spectrometer (ICP, PerkinElmer Optima-5300 DV, USA). The internalized gold concentrations were calculated as the ratio of ng Au per ng protein based on the above results. The ICP measurements were repeated 3 times.

### *In vitro* cell viability assays

To investigate the phototoxicity of GBPs@l-HA and GBPs@h-HA in the cell culture system, cells (MDA-MB-231/Luc, HEK 293T cells) were seeded in 96-well plates at a density of 1 × 10^4^ cells per well. After 24 h of growth, various concentrations of the nanoparticles (ranging from 1.875 mg L^−1^ to 15 mg L^−1^) were added to the culture medium. After incubation for another 24 h, 5 mg L^−1^ of MTT solution was diluted 10 times in the culture medium, and 100 μL of the diluted MTT solution was added to each well. After 4 h of incubation at 37 °C, the medium was discarded, and 150 μL of DMSO was added for approximately 20 min to lyse the cells by gently stirring and solubilising the formazan crystals. The toxicity of the samples was evaluated *via* the absorbance measured with a microplate reader (Tecan, Infinite 200, Australia) at 490 nm. To investigate the photothermal effect of GBPs@l-HA and GBPs@h-HA, the same cells incubated with the nanoparticles were irradiated with an 808 nm laser for 7 min at a power density of 1.2 W cm^−2^, and the MTT assays were performed next. The cell viability assays were calculated according to our previous work^41^ and repeated 3 times.

### *In vivo* photothermal therapeutic efficacy and biocompatibility

All animal procedures were performed in accordance with the Guidelines for Care and Use of Laboratory Animals of Jinling Hospital and approved by the Animal Ethics Committee of Jinling Hospital. Subcutaneous tumours were created by subcutaneous injection of MDA-MB-231/Luc cells into the back of each mouse. The mice with tumours with similar diameters of 5–8 mm were divided into three groups, including the therapy groups and the control group. For the PTT treatment with GBPs@l-HA and GBPs@h-HA, the power of 808 nm NIR laser was selected to be 1.2 W cm^−2^ for 7 min, and thermal images were captured during irradiation by an infrared camera (MAGNITY f15F1, Wuhan VST Light & Technology Co., Ltd., China). The bioluminescent images of the mice were obtained using an IVIS Lumina imaging system (Caliper, USA) before and two days after each treatment. The average bioluminescent signal intensity measured and normalized by the software (Caliper, USA) reflects the trend of the tumour progression. The tumour volume (tumour length × tumour width) and body weight of the mice were recorded at each time point during the following 14 days; after that, the mice were sacrificed. The major organs including heart, liver, spleen, lung, and kidney as well as the tumour were collected for haematoxylin and eosin (H&E) staining to observe pathological changes in them.

### Hemolysis assay

Fresh whole blood samples were provided by Jinling Hospital. Red blood cells (RBCs) were collected by centrifugation and were washed with ice-cold saline three times. The resuspended RBCs were then incubated with GBPs, GBPs@l-HA and GBPs@h-HA at concentrations ranging from 1.875 to 15 mg L^−1^ for 2 h at 37 °C, compared with RBCs incubated with ultrapure water (positive control) and saline (negative control). The haemolysis assay was performed following the described protocol in our previous report, and the results were repeated 3 times.^42^

### Statistical analysis

All statistical comparisons were performed using Student's *t*-test. Data were expressed as the mean ± SD. A two-sided *p*-value of less than 0.05 was considered significant in all of the statistical tests.

## 3 Results and discussion

**Fig. 1:**
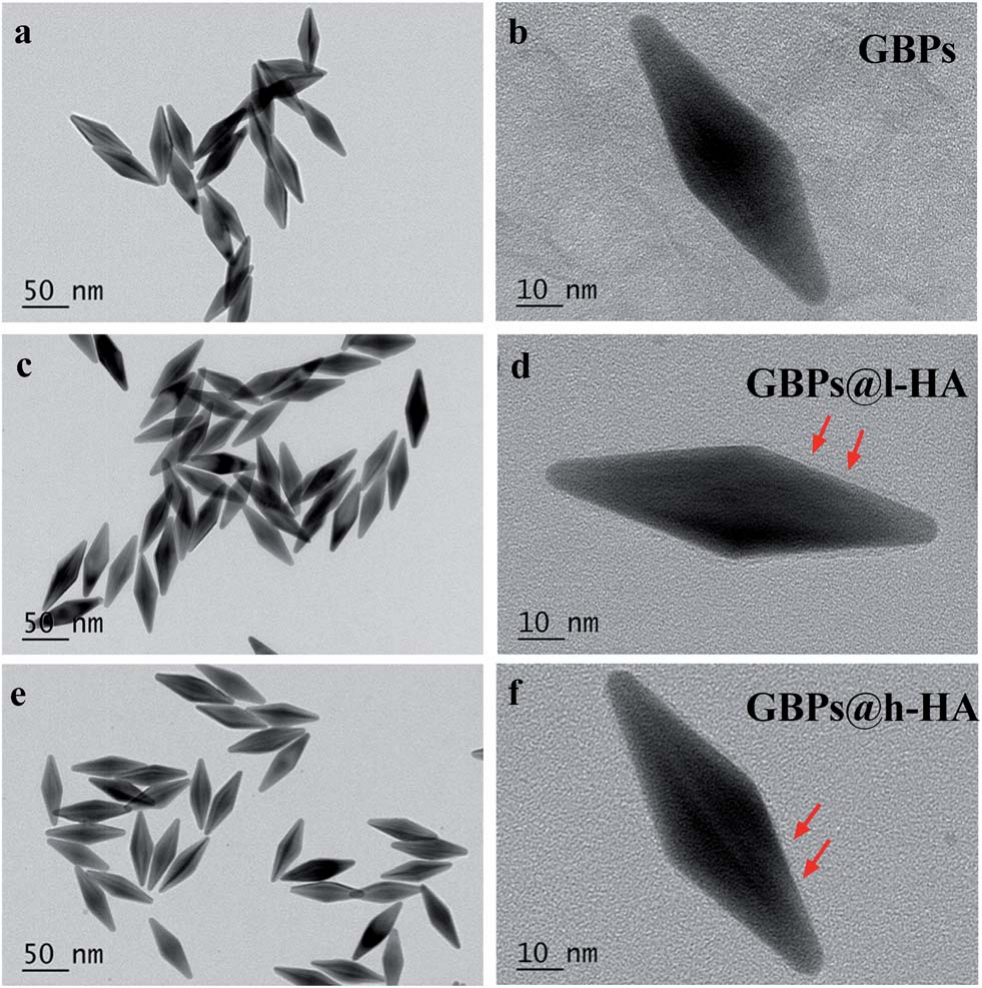
TEM images of (a and b) GBPs (c and d) GBPs@l-HA and (e and f) GBPs@h-HA (scale bars: 50 nm and 10 nm). Red arrows show the l-HA or h-HA forming the shells of GBPs.

**Fig. 2:**
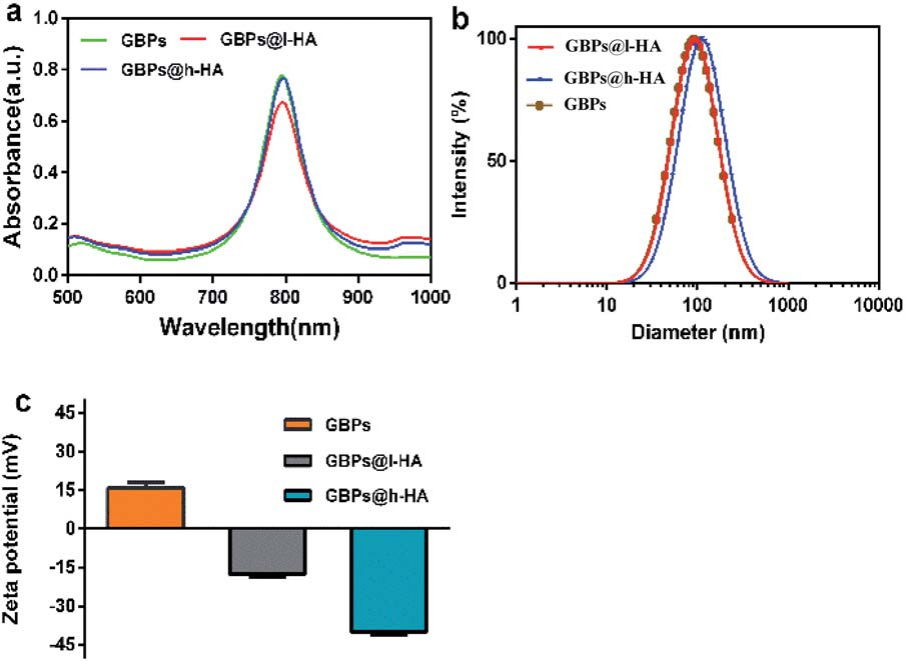
(a) UV-vis spectra of GBPs, GBPs@l-HA, and GBPs@h-HA. (b) The hydrodynamic diameter of the GBPs, GBPs@l-HA, and GBPs@h-HA. (c) Zeta potential of the GBPs, GBPs@l-HA, and GBPs@h-HA.

The nude GBPs were first fabricated by stepwise seed-mediated growth as previously reported.^40,43^ Then, the appropriate amount of GBPs without CTAB was added to get a bilayer with l-HA or h-HA around the surface. As evidenced by the TEM images (Fig. 1d and f), the GBPs@l-HA or GBPs@h-HA possess outer shells of a similar thickness (2.549 ± 0.054 and 2.385 ± 0.109 nm), suggesting the l-HA or h-HA molecules were successfully loaded on the GBPs. In addition, GBPs, GBPs@l-HA and GBPs@h-HA have nearly the same length (79.375 ± 1.145, 83.091 ± 2.079 and 89.273 ± 1.721 nm, respectively) and width (25.772 ± 1.006, 27.924 ± 2.063 and 28.872 ± 1.382 nm, respectively) (Fig. 1a–f). The characterization of the GBPs@l-HA and GBPs@h-HA fabrication was investigated by UV-vis spectroscopy, dynamic light scattering (DLS) and a Nano-Zetasizer (Zeta). The characteristic absorption peak of GBPs@l-HA and GBPs@h-HA at 795 nm was found in the UV-vis spectrum of GBPs (Fig. 2a). The HA that covalently bound to GBPs did not change the basic physical and chemical properties of the GBPs. In this study, the DLS measurements revealed the hydrodynamic diameter of GBPs, GBPs@l-HA and GBPs@h-HA was 70.7 ± 1.3 nm, 93.9 ± 0.9 nm and 112.1 ± 1.0 nm, respectively (Fig. 2b). As expected, the zeta potential measurement showed that GBPs@l-HA and GBPs@h-HA displayed a negative surface charge of −17.69 ± 1.2 mV and −39.76 ± 1.45 mV, respectively, owing to the modification of the anionic polymer HA on the surface of the GBPs (Fig. 2c). The HA density on the surface of the GBPs affects their cellular binding and targeting ability.^25^ To precisely quantify the conjugation efficiency, we took the CTAB precipitation method to analyse the HA concentration, and the mass ratio of l-HA and h-HA to GBPs was 13.22 and 4.77, respectively. The higher conjugation density induced a faster clearance *in vivo* for their higher binding affinity to macrophages and other receptors.^24,25,44^

**Fig. 3:**
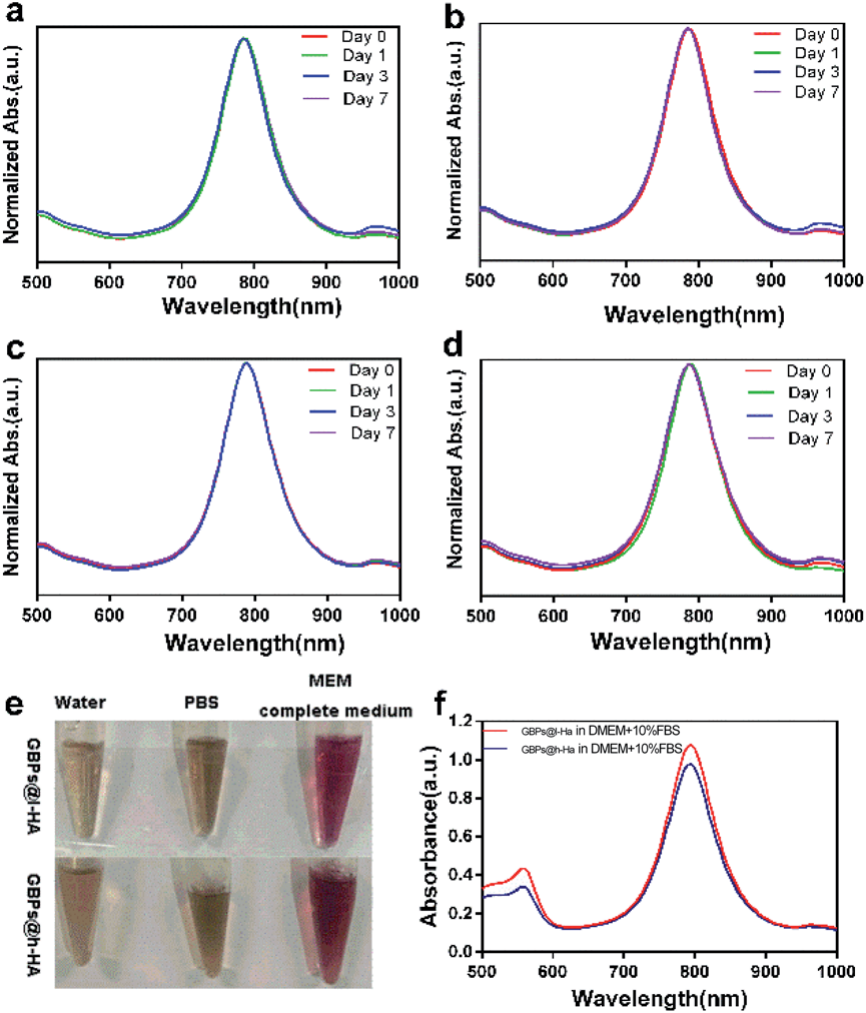
(a–d) UV-vis spectra of GBPs@l-HA or GBPs@h-HA dispersed in water (a and c) and PBS (pH 7.4) (b and d) solution for different time periods. (e and f) The stability of GBPs@l-HA and GBPs@h-HA in MEM.

To determine the colloidal stability of the GBPs@l-HA and GBPs@h-HA in aqueous condition, the LSPR peaks of the above nanostructures were monitored by a UV-vis spectrometer.^45,46^Fig. 3a–d shows the absorption spectra of GBPs@l-HA or GBPs@h-HA dispersed in water and PBS (pH 7.4) solution for different time periods. After standing for 7 days, the LSPR bands of GBPs@l-HA and GBPs@h-HA and their intensities remained unchanged, indicating their excellent stability in aqueous solution. Additionally, GBPs@l-HA and GBPs@h-HA had excellent stability in MEM (Fig. 3e and f).

**Fig. 4:**
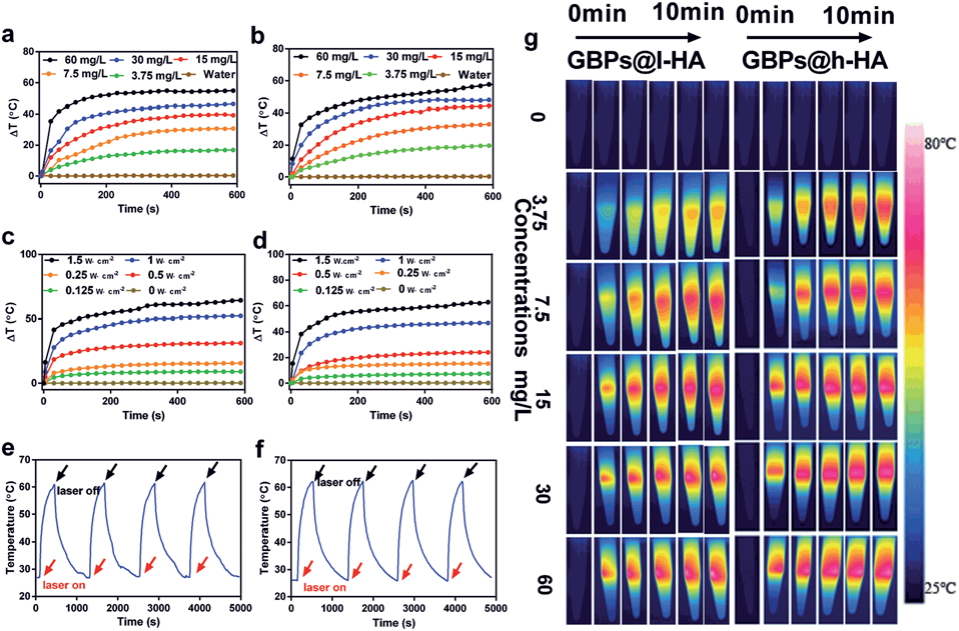
(a and b) Temperature elevation of GBPs@l-HA and GBPs@h-HA at different concentrations (corresponding to 3.75, 7.5, 15, 30 and 60 mg L^−1^ of GBPs) during 10 min irradiation (808 nm, 1.2 W cm^−2^). (c and d) Temperature elevation of GBPs@l-HA and GBPs@h-HA (corresponding to 60 mg L^−1^ of GBPs) under irradiation at different power densities (0, 0.125, 0.25, 0.5, 1.0, 1.5 W cm^−2^) during 10 min irradiation (808 nm). (e and f) Temperature changes of GBPs@l-HA and GBPs@h-HA (60 mg L^−1^) during four irradiation cycles (irradiating by 808 nm laser at 0.9 W cm^−2^ for 3 min and cooling for 7 min). (g) Photographs of different concentrations of GBPs@l-HA and GBPs@h-HA irradiated at 1.2 W cm^−2^ for 10 min.

The photothermal performance of the GBPs@l-HA and GBPs@h-HA was investigated, and the temperatures of the aqueous solutions increased along with the nanoparticle concentrations. The temperatures of GBPs@l-HA and GBPs@h-HA solution (60 mg L^−1^) rapidly increased by 54.9 °C and 57.8 °C, respectively, upon irradiation with an 808 nm laser at 1.2 W cm^−2^ for 10 min. In contrast, a minor temperature increase in water was detected (Fig. 4a, b and g). In short, independent of the MW of HA, the GBPs@h-HA and GBPs@l-HA exhibited similar photothermal performance. As shown in Fig. 4c and d, the temperatures of GBPs@l-HA (60 mg L^−1^) and GBPs@h-HA (60 mg L^−1^) increased by 31.3 °C and 24.04 °C at 0.5 W cm^−2^ for 10 min and 64.3 °C and 62.7 °C at 1.5 W cm^−2^ for 10 min, suggesting that the photothermal effect of GBPs@l-HA and GBPs@h-HA has no significant differences from each other at higher heating power. It has been illustrated that temperatures above 60 °C activate cellular degradation pathways, such as protein denaturation, folding and cross-linking of DNA, generating instantaneous coagulative necrosis and irreversible tumour cell death.^47^ Additionally, GBPs@l-HA and GBPs@h-HA dispersions exhibit good photothermal conversion ability after four irradiation cycles (irradiated by an 808 nm laser at 0.9 W cm^−2^ for 3 min and cooling for 7 min). On the basis of these data, both nanoparticles present good photostability and have the potential to be used as photothermal agents for cancer treatment (Fig. 4e and f).

**Fig. 5:**
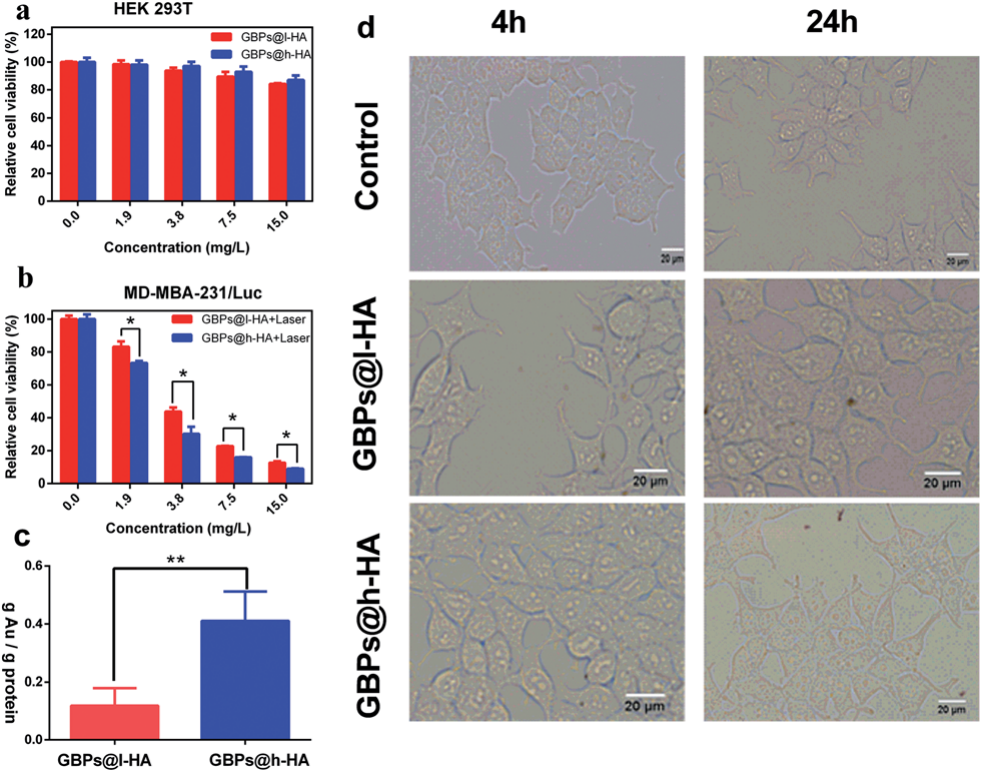
(a) Relative viability of HEK 293T cells incubated with GBPs@l-HA and GBPs@h-HA for 24 h. (b) Relative viability of MDA-MB-231/Luc cells incubated with GBPs@l-HA and GBPs@h-HA for 24 h, following by 808 nm laser irradiation (1.2 W cm^−2^, 5 min, *: *p* < 0.05). The error bars stand for the standard deviations. (c) The gold uptake of MDA-MB-231/Luc cells quantified by inductively coupled plasma after incubating with GBPs@l-HA and GBPs@h-HA 24 h (**: *p* < 0.01). (d) Cell morphology of HEK 293T cells incubated with GBPs@l-HA or GBPs@h-HA for 4 h and 24 h using the Olympus microscope. Scale bar = 20 μm.

To further confirm the biocompatibility and therapeutic efficiency of the nanoparticles, GBPs@l-HA or GBPs@h-HA at various concentrations (from 1.875 mg L^−1^ to 15 mg L^−1^) were incubated with HEK 293T cells for 24 h. Then, MTT assays were performed to detect the cell viability. The cell viability remained greater than 84% at a nanoparticle concentration of 15 mg L^−1^, and no obvious morphology change was captured using the Olympus microscope at 4 h and 24 h (Fig. 5a and d), suggesting the low toxicity and excellent biocompatibility of GBPs@l-HA and GBPs@h-HA. When the cells treated with GBPs@l-HA or GBPs@h-HA (ranging from 1.875 mg L^−1^ to 15 mg L^−1^) were irradiated by an 808 nm laser at a power density of 1.2 W cm^−2^ for 5 minutes, GBPs@l-HA and GBPs@h-HA exhibited a concentration-dependent photothermal therapeutic effect (Fig. 5b). As shown in Fig. 5b, GBPs@h-HA induced more cancer cell death compared to GBPs@l-HA at the corresponding concentration. These cell viability data clearly delineated that GBPs@h-HA was much more efficacious than GBPs@l-HA in terms of therapeutic efficiency but had a similar biocompatibility in normal cells.

To illustrate the causation of the different therapeutic efficacies, inductively coupled plasma atomic emission spectrometry (ICP-AES) was chosen to evaluate the cellular uptake of GBPs@l-HA and GBPs@h-HA (Fig. 5c). Briefly, the ratio between the amount of Au and protein was used to calculate the intracellular concentration of gold. After incubation with MDA-MB-231/Luc cells for 24 h, GBPs@h-HA (0.41 ± 0.1 ng Au per ng protein) exhibited more uptake than GBPs@l-HA incubated (0.1185 ± 0.0626 ng Au per ng protein). Because different molecular weight HA has quite different binding affinity to CD44 receptors, our results showed that cellular uptake of GBPs@h-HA was 3.46 times higher compared to GBPs@l-HA. However, the difference of GBPs@h-HA and GBPs@l-HA in the relative cell viability was not as much as that in ICP analysis. Due to the 808 nm NIR irradiation (1.2 W cm^−2^, 5 min) in a relative cell viability assay, the GBPs@l-HA could raise the temperature, and the temperature was high enough to cause massive cell death.

Our results revealed that GBPs@h-HA had higher cellular uptake compared with GBPs@l-HA. It is known that hyaluronic acid can actively target the CD44 receptor, which is overexpressed in triple-negative breast cancer and the other solid tumours.^25,48^ As a major receptor of HA, coating the nanoparticles with HA could increase the cellular uptake of HA-based nanoagents.^24,25^ The coated nanoparticles presumably enter the cytosol *via* receptor-mediated endocytosis, which could be affected by the binding affinity between HA and CD44 receptors.^49–51^ In H. S. S. Qhattal's research, the binding affinity of fluorescein-labelled HA (testing HA binding function) to CD44 receptors was highly dependent on the molecular weight of the HA; higher MW HA had a higher binding affinity than that of lower MW HA.^25^ H. S. S. Qhattal *et al.* further modified liposomes with different MW HA and exhibited that by increasing the MW of HA, the cellular uptake of HA-based liposomes could be enhanced, which was consistent with our results. These results illustrated that the molecular weight of HA influences the binding affinity of HA to CD44 receptors, which could further affect the cellular uptake of HA-modified nanoparticles in CD44-positive cells.^24,25^

Compared with PEG modification, HA modification could prolong the blood circulation time to present the obtained nanoagent with stealthiness in the blood system.^24,52^ Xu *et al.* also demonstrated that HA-coated GNRs showed higher blood circulation retention (approximately 8.1% ID) in comparison with reported PEG-, CS- and SiO_2_-decorated GNRs (≤3.0% ID).^45,53–56^

**Fig. 6:**
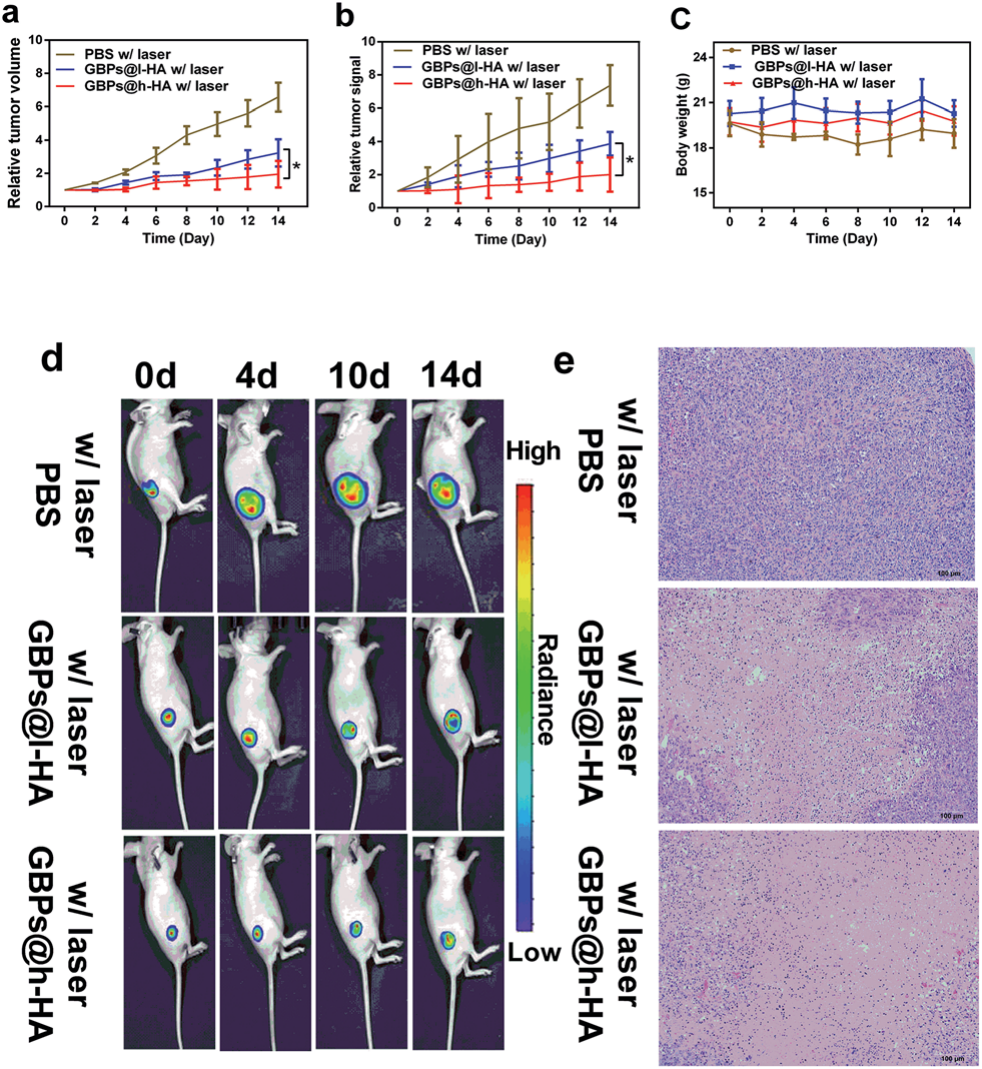
(a) Tumour growth curves of mice in the course of treatment. (b) Relative tumour bioluminescent signal of mice in the course of treatment. The error bars stand for the standard deviations (*n* = 5–7). (c) Body weight of mice from three groups in the period of treatment. The error bars stand for the standard deviations (*n* = 5–7). (d) Bioluminescence images of mice in the course of treatment. (e) H&E staining images of resected tumour sections from the mice (scale bar: 100 μm).

Wu *et al.* demonstrated that HA-coated PLGA nanoparticulate docetaxel can effectively target and suppress human lung cancer *in situ*.^57^ To elucidate the tumour-targeting therapeutic performance of the GBPs@h-HA and GBPs@l-HA in MDA-MB-231/Luc xenografts, MDA-MB-231/Luc-bearing mice were randomly divided into 3 groups and intravenously injected with 100 μL of PBS (pH 7.4), GBPs@l-HA (7.5 mg L^−1^) or GBPs@h-HA solution (7.5 mg L^−1^) and then were irradiated by a laser (808 nm, 1.2 W cm^−2^) for 7 min 24 h after the injections. In the PBS group, it was found that the tumours grow at a fast rate, and the tumour volume increased to nearly 7-fold larger than the initial size by fourteen days post-treatment. Compared with the PBS group, both the GBPs@l-HA group and GBPs@h-HA group showed superior therapeutic performance in inhibiting tumour growth. The relative bioluminescence signal from GBPs@l-HA- and GBPs@h-HA-treated tumours, which was significantly weaker than that from PBS group, also illustrated the better therapeutic performance of the nanoparticles. Moreover, for the GBPs@l-HA group, it was found that the tumours grow at a faster growth rate than GBPs@h-HA group, which was approximately 3.2-fold larger than the initial sizes. By contrast, the tumour volumes of GBPs@h-HA group were respectively smaller than that of the GBPs@l-HA group and approximately 2.0-fold larger than the initial size. These results indicated that the HA-coated gold nanobipyramids, no matter if it was 102 kDa or 380 kDa, both decreased the growth rate of tumours to some extent. Moreover, the higher molecular weight of HA-coated gold nanobipyramids, GBPs@h-HA, provided a much superior effect than GBPs@l-HA in tumour growth inhibition (Fig. 6a), which was consistent with the results revealed by the relative bioluminescence signal curve (Fig. 6b). Additionally, no obvious loss of body weight was observed in any of the three groups during the treatment period (Fig. 6c). Bioluminescence images of MDA-MB-231/Luc tumours before and two days after each laser treatment were recorded over 14 days, and the representative images were summarized in Fig. 6d. A remarkably lower signal was observed from GBPs@h-HA-treated tumours when compared with that from GBPs@l-HA-treated ones, which was in accordance with the results from the abovementioned tumour growth curves. Taking these results together, it is concluded that h-HA (380 kDa)-coated gold nanobipyramids possess more significant therapeutic efficacy *in vivo*.

H&E images of tumours also revealed the intensive necrosis in the entire area of the tumour centre from the GBPs@h-HA-treated group, while residual malignant cells were observed in tumours treated by GBPs@l-HA (Fig. 6e). As expected, no obvious necrosis was observed in tumours treated with PBS with irradiation, implying that intravenously injected targeted treating nanoparticles can be operated as a powerful tumour growth suppressing agent in *in vivo* therapy of triple-negative breast cancer.

**Fig. 7:**
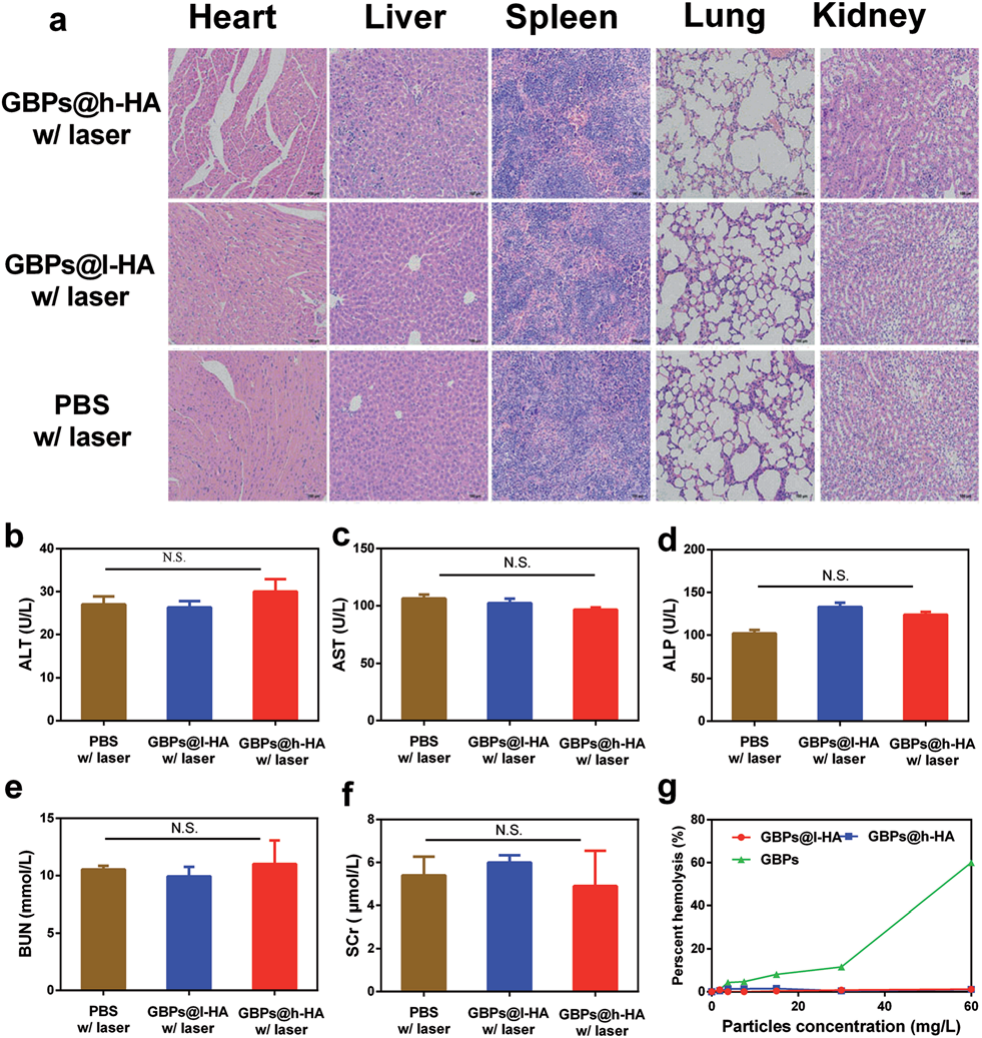
(a) H&E staining of major organ sections excised from MDA-MB-231/Luc human breast tumour-bearing mice following 14 d of treatment with PBS plus irradiation, GBPs@l-HA plus irradiation and GBPs@h-HA plus irradiation (the images were captured at high magnification (400×)). (b–f) Blood biochemical indexes (ALT, AST, ALP, BUN, and SCr) of mice post-treatment. (g) Percentage of red blood cell haemolysis incubated with GBPs, GBPs@l-HA, and GBPs@h-HA at different concentrations (0, 3.75, 7.5, 15.30 and 60 mg L^−1^).

H&E-stained slices of organ sections (heart, liver, spleen, lung, and kidney) did not reveal any significant tissue damage in the GBPs@l-HA- or GBPs@h-HA-treated groups compared with the PBS group (Fig. 7a). Furthermore, blood examination indicated no significant difference among the 3 groups among the indicators of liver injury, such as alanine aminotransferase (ALT), aspartate aminotransferase (AST), alkaline phosphatase (ALP), and other renal biochemical indexes, such as blood urea nitrogen (BUN) and serum creatinine (SCr) (Fig. 7b–f). These data revealed that the liver and renal function of mice were not injured after being treated with GBPs@l-HA or GBPs@h-HA. Moreover, the haemolytic activities of GBPs@h-HA and GBPs@l-HA were measured to be as low as 1.2%, far lower than that of GBPs (60%), even at particle concentrations up to 60 mg L^−1^, which further illustrated the fascinating biocompatibility of GBPs@h-HA and GBPs@l-HA (Fig. 7g). Collectively, the histological and serum analyses indicated the negligible toxicity and reliable biosafety of GBPs@h-HA and GBPs@l-HA on animals over a long period of monitoring, confirming their potential for further clinical applications.

## 4 Conclusion

In this report, we compared the cellular uptake and therapeutic efficacy between gold nanobipyramids coated by high (380 kDa) and low (102 kDa) molecular weight HA. ICP studies showed GBPs@h-HA possesses a higher cellular uptake in MDA-MB-231 breast cancer cells when compared with GBPs@l-HA and is able to enhance photothermal therapeutic efficacy both *in vitro* and *in vivo*. Overall, our results demonstrate that the molecular weight of HA plays an important role in the targeting ability of HA-coated gold nanobipyramids and may provide insights for selecting the optimal MW of HA to maximize the therapeutic efficacy of CD44-targeted photothermal therapy.

## Conflicts of interest

There are no conflicts to declare.

## Supplementary Material

## References

[cit1] DeSantis C. E., Fedewa S. A., Goding Sauer A., Kramer J. L., Smith R. A., Jemal A. (2016). Ca-Cancer J. Clin..

[cit2] Kesharwani P., Ghanghoria R., Jain N. K. (2012). Drug Discovery Today.

[cit3] Jain A., Jain K., Kesharwani P., Jain N. K. (2013). J. Nanopart. Res..

[cit4] Foulkes W. D., Smith I. E., Reisfilho J. S. (2010). N. Engl. J. Med..

[cit5] Dent R., Trudeau M., Pritchard K. I., Hanna W. M., Kahn H. K., Sawka C. A., Lickley L. A., Rawlinson E., Sun P., Narod S. A. (2007). Clin. Cancer Res..

[cit6] Lin N. U., Vanderplas A., Hughes M. E., Theriault R. L., Edge S. B., Wong Y. N., Blayney D. W., Niland J. C., Winer E. P., Weeks J. C. (2012). Cancer.

[cit7] Liedtke C., Mazouni C., Hess K. R., André F., Tordai A., Mejia J. A., Symmans W. F., Gonzalezangulo A. M., Hennessy B., Green M. (2008). J. Clin. Oncol..

[cit8] Gucalp A., Traina T. A. (2011). Chemother. Res. Pract..

[cit9] Zheng Z., Shao N., Weng H., Li W., Zhang J., Zhang L., Yang L., Ye S. (2015). Med. Oncol..

[cit10] Olsson E., Honeth G., Bendahl P. O., Saal L. H., Gruvberger-Saal S., Ringner M., Vallon-Christersson J., Jonsson G., Holm K., Lovgren K., Ferno M., Grabau D., Borg A., Hegardt C. (2011). BMC Cancer.

[cit11] Lo Y. L., Wang Y. S., Wang L. F. (2013). Adv. Healthcare Mater..

[cit12] https://cgap.nci.nih.gov/SAGE

[cit13] Zoller M. (2011). Nat. Rev. Cancer.

[cit14] Xu H., Tian Y., Yuan X., Liu Y., Wu H., Liu Q., Wu G. S., Wu K. (2016). OncoTargets Ther..

[cit15] Toole B. P. (2009). Clin. Cancer Res..

[cit16] Misra S., Heldin P., Hascall V. C., Karamanos N. K., Skandalis S. S., Markwald R. R., Ghatak S. (2011). FEBS J..

[cit17] Chen X., Liu Z., Parker S. G., Zhang X., Gooding J. J., Ru Y., Liu Y., Zhou Y. (2016). ACS Appl. Mater. Interfaces.

[cit18] Mero A., Campisi M., Caputo M., Cuppari C., Rosato A., Schiavon O., Pasut G. (2015). Curr. Drug Targets.

[cit19] Mattheolabakis G., Milane L., Singh A., Amiji M. M. (2015). J. Drug Targeting.

[cit20] Wolny P. M., Banerji S., Gounou C., Brisson A. R., Day A. J., Jackson D. G., Richter R. P. (2010). J. Biol. Chem..

[cit21] D'Agostino A., Stellavato A., Corsuto L., Diana P., Filosa R., La G. A., De R. M., Schiraldi C. (2017). Carbohydr. Polym..

[cit22] Delpech B., Girard N., Bertrand P., Courel M. N., Chauzy C., Delpech A. (1997). J. Intern. Med..

[cit23] Lennon F. E., Mirzapoiazova T., Mambetsariev N., Mambetsariev B., Salgia R., Singleton P. A. (2014). J. Biol. Chem..

[cit24] Qhattal H. S. S., Hye T., Alali A., Liu X. (2014). ACS Nano.

[cit25] Qhattal H. S., Liu X. (2011). Mol. Pharm..

[cit26] Mizrahy S., Raz S. R., Hasgaard M., Liu H., Soffer-Tsur N., Cohen K., Dvash R., Landsman-Milo D., Bremer M. G., Moghimi S. M. (2011). J. Controlled Release.

[cit27] Vigderman L., Khanal B. P., Zubarev E. R. (2012). Adv. Mater..

[cit28] Chen H., Shao L., Li Q., Wang J. (2013). Chem. Soc. Rev..

[cit29] Li Q., Zhuo X., Li S., Ruan Q., Xu Q. H., Wang J. (2015). Adv. Opt. Mater..

[cit30] Kou X., Ni W., Tsung C. K., Chan K., Lin H. Q., Stucky G. D., Wang J. (2007). Small.

[cit31] Zhang H., Liu J. (2016). Opt. Lett..

[cit32] Liu W., Liu D., Zhu Z., Han B., Gao Y., Tang Z. (2014). Nanoscale.

[cit33] Chen X., Liu Z., Parker S. G., Zhang X., Gooding J. J., Ru Y., Liu Y., Zhou Y. (2016). ACS Appl. Mater. Interfaces.

[cit34] Deng H., Dai F., Ma G., Zhang X. (2015). Adv. Mater..

[cit35] Zhang Z., Wang J., Chen C. (2013). Adv. Mater..

[cit36] Zheng M., Yue C., Ma Y., Gong P., Zhao P., Zheng C., Sheng Z., Zhang P., Wang Z., Cai L. (2013). ACS Nano.

[cit37] Zhu A., Miao K., Deng Y., Ke H., He H., Yang T., Guo M., Li Y., Guo Z., Wang Y. (2015). ACS Nano.

[cit38] Zhang Z., Wang L., Wang J., Jiang X., Li X., Hu Z., Ji Y., Wu X., Chen C. (2012). Adv. Mater..

[cit39] Fillmore C., Kuperwasser C. (2007). Breast Cancer Res..

[cit40] Guo Z., Wan Y., Wang M., Xu L., Lu X., Yang G., Fang K., Gu N. (2012). Colloids Surf., A.

[cit41] Wang S., Teng Z., Huang P., Liu D., Liu Y., Tian Y., Sun J., Li Y., Ju H., Chen X., Lu G. (2015). Small.

[cit42] Ma X., Cheng Y., Huang Y., Tian Y., Wang S., Chen Y. (2015). RSC Adv..

[cit43] Wang S., Ma X., Hong X., Chen Y., Tian Y., Zhao S., Liu W., Tang Y., Zhao R., Song L., Teng Z., Lu G. (2018). ACS Nano.

[cit44] Mcneeley K. M., Annapragada A., Bellamkonda R. V. (2007). Biochem. Syst. Ecol..

[cit45] Xu W., Qian J., Hou G., Suo A., Wang Y., Wang J., Sun T., Yang M., Wan X., Yao Y. (2017). ACS Appl. Mater. Interfaces.

[cit46] Robinson J. T., Tabakman S. M., Liang Y., Wang H., Casalongue H. S., Vinh D., Dai H. (2011). J. Am. Chem. Soc..

[cit47] Jang B., Park J. Y., Tung C. H., Kim I. H., Choi Y. (2011). ACS Nano.

[cit48] Naor D., Sionov R. V., Ish-Shalom D. (1997). Adv. Cancer Res..

[cit49] Qiu L., Li Z., Qiao M., Long M., Wang M., Zhang X., Tian C., Chen D. (2014). Acta Biomater..

[cit50] Son G. M., Kim H. Y., Ryu J. H., Chu C. W., Kang D. H., Su B. P., Jeong Y. I. (2014). Int. J. Mol. Sci..

[cit51] Li J., Huo M., Wang J., Zhou J., Mohammad J. M., Zhang Y., Zhu Q., Waddad A. Y., Zhang Q. (2012). Biomaterials.

[cit52] Zhang Q., Deng C., Fu Y., Sun X., Gong T., Zhang Z. (2016). Mol. Pharm..

[cit53] Black K. C. L., Wang Y., Luehmann H. P., Cai X., Xing W., Pang B., Zhao Y., Cutler C. S., Wang L. V., Liu Y. (2014). ACS Nano.

[cit54] Zhang Z., Wang J., Nie X., Wen T., Ji Y., Wu X., Zhao Y., Chen C. (2014). J. Am. Chem. Soc..

[cit55] Chen R., Wang X., Yao X., Zheng X., Wang J., Jiang X. (2013). Biomaterials.

[cit56] Peer D., Margalit R. (2004). Int. J. Cancer.

[cit57] Wu J., Deng C., Meng F., Zhang J., Sun H., Zhong Z. (2016). J. Controlled Release.

